# Salivary glands require Aurora Kinase B for regeneration after transient innate immune-mediated injury

**DOI:** 10.1038/s41598-019-47762-9

**Published:** 2019-08-05

**Authors:** Abeer Shaalan, Gordon Proctor

**Affiliations:** Centre for Host-Microbiome Interactions, Faculty of Dentistry, Oral & Craniofacial Sciences, King’s College London, London, United Kingdom

**Keywords:** Salivary gland diseases, Mitosis, Toll-like receptors

## Abstract

Severe, irreversible salivary gland disease and oral dryness is experienced by sufferers of Sjögren’s syndrome and those treated with irradiation for head and neck cancer. Therefore, major efforts have been made in the last decade to unravel key molecular signals that can drive salivary gland (SG) regeneration and functional restoration. However, the earliest molecular determinants that accompany SG regeneration remain incompletely defined. The present study examined the initial mitogenic events marking the regenerative response of the murine submandibular gland (SMG), following innate immune-mediated injury. Local intraductal administration of the synthetic double stranded (ds) RNA polyinosinic-polycytidylic acid (poly (I:C)) widely, but transiently, depleted the acinar and progenitor cells, 24 hours post poly (I:C) introduction. While the progenitor and duct cells started to proliferate and expand at 72 hours, the Mist1-positve acinar cells did not re-appear until 96 hours post poly (I:C) injury. The cellular replenishment during regeneration involved significant upregulation of the cell cycle promoter Aurora kinase B (AURKB). AURKB, which is expressed in healthy proliferating and cancerous cells, is a serine/threonine protein kinase, well known to orchestrate key events in cell division and cytokinesis. However, the expression and role of AURKB in regeneration of post mitotic salivary gland cells has not been previously explored. *In vivo* inhibition of AURKB using the selective inhibitor Barasertib (AZD1152-HQPA) interfered with SMG recovery from the transient, but severe poly (I:C)-mediated injury and cellular depletion. AURKB deficiency during regeneration of the injured tissues: disrupted cell cycle progression, repressed renewal of Mist1-positive acinar cells and prevented recovery of salivary secretion. The knowledge gained in this study may be utilized in the development of therapeutic targets for irreversible salivary gland disease.

## Introduction

The earliest events that accompany salivary gland regeneration remain incompletely defined. A novel salivary gland injury/regeneration model was recently developed, based on local activation of the submandibular gland (SMG) innate immunity^1^. The C57BL/6 SMG was immunologically challenged by local intraductal infusion of the synthetic ds RNA: poly (I:C). The initial 24 hours following innate immune activation comprised the injury phase and featured: (i) acute loss of saliva production^1^, (ii) TLR3-mediated apoptosis, inflammation and cytokine production^2^, (iii) generation of reactive nitrogen species (RNS) and disruption of intracellular calcium^3^. The aim of the present study was to characterise early events in regeneration of the SMGs, following the transient innate immune-mediated injury.

Within the regeneration context, complex structures depend on the availability of cells (whether a stem cell reservoir or injury-reactivated differentiated cell) to undergo significant proliferation and re-development of the missing tissues, followed by activation of specific differentiation programs^4^. In the current model, the acinar compartment was considerably depleted, except for a population of proliferating Mist1-positive cells. Intriguingly, and only after 24 hrs of this structural loss, the SMG was re-populated with proliferating and non-proliferating acinar cells.

Importantly, following up on our PCR array finding (unpublished), which highlighted the significant upregulation of the cell cycle-associated aurora kinase B or AURKB, revealed approximately 25-fold increase in its transcription level during early regeneration. AURKB, which has not yet been described in any model of salivary gland regeneration, has been recently reported to be indispensable during neuronal regeneration. Indeed, AURKB pharmacologic inhibition delayed neurite extension and recovery following UV laser-mediated injury^5^. Here, we used the Aurora B small molecule inhibitor, AZD1152-HQPA, to selectively downregulate Aurora B activity^6^ and investigate its role during the initial exocrine regeneration phase.

In the present study, we show for the first time that AURKB pharmacological inhibition *in vivo* interfered with early salivary gland regeneration following poly (I:C) injury. This included: (i) disrupted cell cycle progression, (ii) sustained depletion of Mist1-positive acinar cells and (iii) lack of functional restoration.

## Results

### Poly (I:C) induced loss and rapid regain of SMG secretion

**Figure 1:**
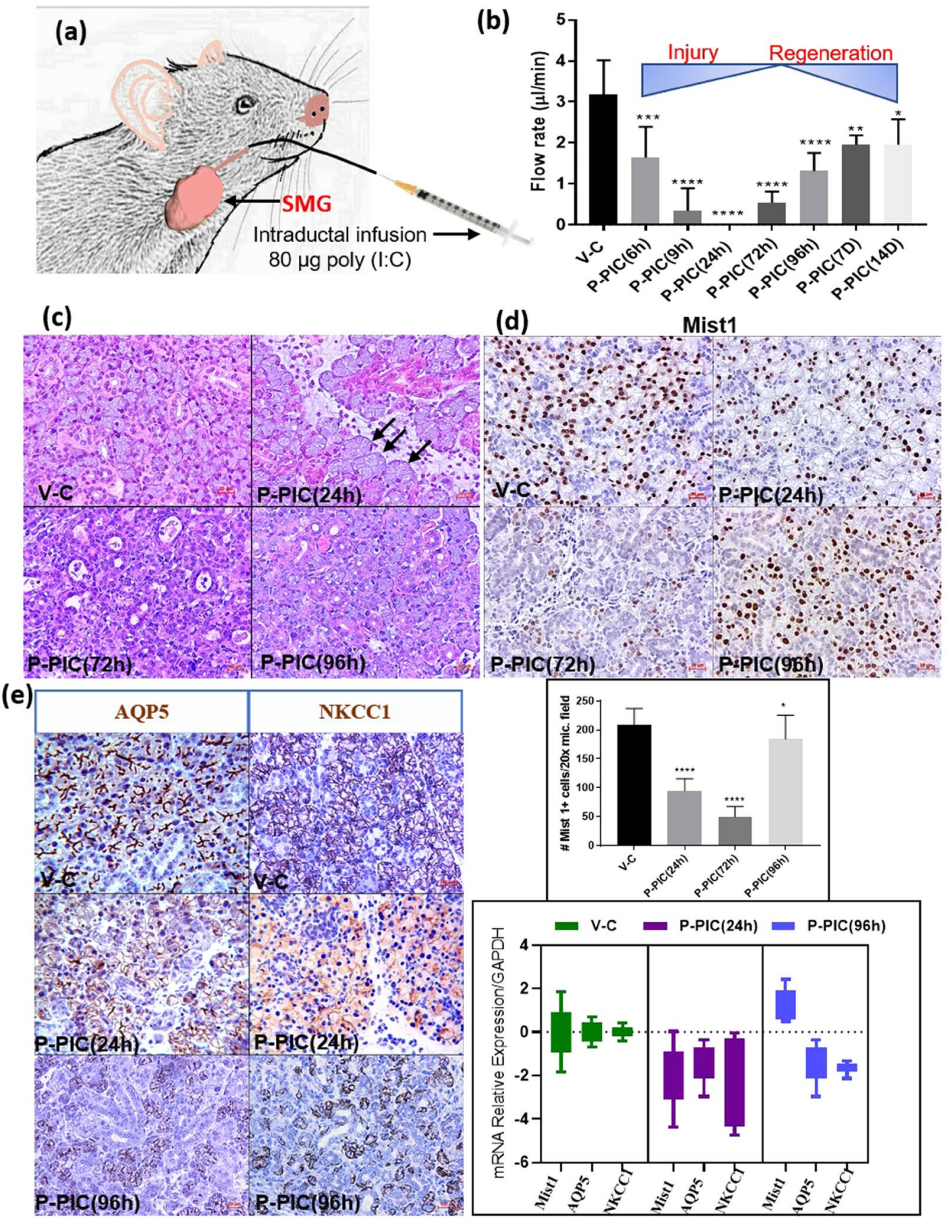
Acute injury/regeneration SG model: (**a**) Illustration depicting poly (I:C) retrograde ductal injection: 20 µl of poly (I:C) (4 µg/µl) was injected via an insulin syringe into the C57BL/6 SMG via Wharton’s duct. (**b**) Measurement of pilocarpine-stimulated saliva: retro-ductal injection of the vehicle (V-C) did not affect SMG saliva flow rate. Conversely, post poly (I:C) (P-PIC), saliva secretion was promptly lost, but was rapidly restored after 72 hrs following the innate immune stimulant. Note the progressive functional recovery after 96 hrs, 7 and 14 days (n = 5–14 gland/group). (**c**) SMG H&E analysis: the control SMGs (V-C) revealed normal compact parenchyma formed of secretory acini and collecting ducts. At 24 hrs, in addition to the inter-and intra-lobular infiltration of immune cells triggered by poly (I:C), acini were enlarged and occasionally devoid of nuclei (arrows). After 72 hrs (initial recovery of salivation), ducts and duct-like structures prevailed in the SMGs, with obvious reduction in the acinar phenotype. The histomorphology dramatically changed at 96 hrs following poly (I:C) injection: newly emerging, basophilic acinar cells were globally seen in the regenerating SMGs. Scale bar = 20 µm. (**d**) Transient loss and rapid regain of the acinar phenotype was confirmed using the acinar differentiation marker Mist1. Approximately, 50% acinar reduction was observed, 24 hrs post innate immune challenge. This prompt depletion became more extensive (80%) at 72 hrs. Surprisingly, after 96 hrs of poly (I:C) challenge, the SMGs were significantly re-populated by Mist1-positive cells, the percentage of which approached the control values (mean ± SD, n = 15 fields (20×)/group, ****P < 0.0001 and *P ≤ 0.05). Scale bar = 20 µm. (**e**) IHC and PCR of AQP5 and NKCC1: extreme loss of these key functional determinants 24 hrs P-PIC and their incomplete recovery at the 96 hrs time point which featured enhanced, control-like expression of acinar Mist1. Scale bar = 20 µm.

Local intraductal injection of the innate immune stimulant poly (I:C) prompted loss of function, which started after 6 hrs following the retrograde injection and declined progressively until the glands ceased secretion completely after 24 hrs. Nevertheless, functional recovery was perceived when saliva was collected 72 hrs following SMG infection and was further enhanced at the following collection time points: 96 hrs, 7 days and 14 days after poly (I:C) injection (Fig. 1b).

### Transient depletion and rapid re-population of the acinar compartment during regeneration

Histologic examination of the SMGs at the peak injury (24 h post poly (I:C)), as well as the initial and peak regeneration phases (72 h and 96 h post poly (I:C), respectively) revealed altered morphology and cellular composition. Local introduction of poly (I:C) triggered an extensive inter- and intralobular inflammatory response. Many of the acinar cells at this time point lacked nuclei and were interspersed in the connective tissue in the form of isolated islands (Fig. 1c). The earliest stage of functional recovery, i.e. 72 hrs post infection, revealed remarkably hypocellular lobules, expansion of the stromal tissues, extensive invasion of immune cells (Supplementary Fig. S1), predominance of ducts and duct like structures and scarcity of acinar cells, which were very sporadically seen as isolated groups (Fig. 1c,d). Indeed, Mist1 immunolabelling revealed extensive acinar cell depletion (~80%) in the poly (I:C)-injected glands, compared to the vehicle-injected control tissues (Fig. 1d). Strikingly, the SMGs were extensively re-populated with Mist1-positive acinar cells over the next 24 hrs, to exhibit a control-like morphology and Mist1 expression (Fig. 1d). To investigate the cause for the loss of Mist1-positive cells, comprehensive characterization of the model during the peak injury phase (6–24 h post poly (I:C)) revealed extremely significant induction of apoptotic death signals in the acinar compartment, 9hrs post injury^2^ (Supplementary Fig. S2). It is worth noting that although the 96 hrs time point showed remarkable emergence of Mist1-positive acini, the flow rates at this time point were still significantly lower than the normal glands (Fig. 1b). To find the reason for these relatively uncoordinated results, IHC and PCR were done and showed that although Mist1-positive, fully differentiated acinar cells were restored after poly (I:C)-induced depletion, they lacked optimum recovery of key functional determinants like the AQP5 and NKCC1 (Fig. 1e).

### Cell proliferation and expansion in the regenerating SMGs

**Figure 2:**
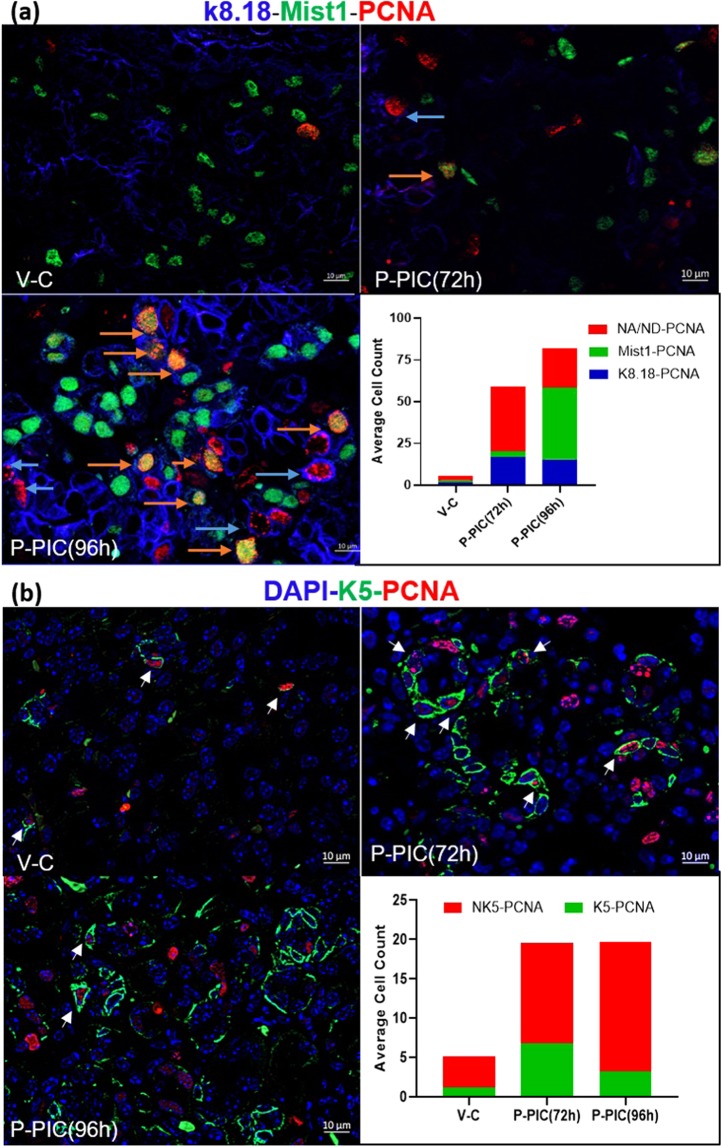
Mist1-K8.18-PCNA, K5-PCNA co-labelling: in general, the control SMGs showed a low rate of cellular turn-over. An overall increase in the number of PCNA-positive cells was seen in the early regeneration phases following poly (I:C) injection (72 and 96 hrs P-PIC) (acinar cells: orange arrows, duct cells: blue arrows). While abundant PCNA co-localization at 72 hrs was seen with K5 progenitor cells (arrows), at 96 hrs the proliferating cells were mostly acini (n > 20 fields from three independent animals: 20x in the Mist1-PCNA-K8.18 experiments and 40x in the K5-PCNA experiments). Scale bar = 10 µm.

One key component in the regeneration of lost complex tissues is the availability of proliferating cells that can differentiate into the cellular make-up of the missing compartments^4^. The excessive and strikingly rapid cellular replenishment seen at 96 hrs, subsequent to the dramatic loss of the acinar phenotype (72 hrs), suggested significant proliferation activity taking place during regeneration, which was further confirmed using PCNA staining (Fig. 2). In the control SMGs, baseline, low-rate proliferation was nearly equally detected in the acinar, ductal and other glandular compartments. At the beginning of functional recovery from poly (I:C) injury (after 72 hrs), the highest cellular expansion was seen in the K5 progenitor compartment, followed by K8.18-positive duct cells. Obviously, at this time point, the expansion rate in the former compartments remarkably exceeded the proliferation activity detected in the residual, sporadically detected acinar cells. However, after 96 hrs, there was a dramatic increase in Mist1-PCNA co-labelled cells at the expense of the duct and progenitor cells (Fig. 2 and Supplementary Fig. S3). To gain insight into other expanding progenitors (non-acinar (NA) and non-ductal (ND), the levels of c-Kit, Sox9 and CK14 cells were assessed during SMG regeneration. A significant increase at the transcriptional and protein levels was seen in the c-kit- and Sox9-positive cells (Supplementary Figs S4 and S5), in addition to increased positivity of CK14 cells, which were very frequently co-localised with Ck5 (Supplementary Fig. S6).

### Poly (I:C)-altered mitogenic signals in the SMGs

**Figure 3:**
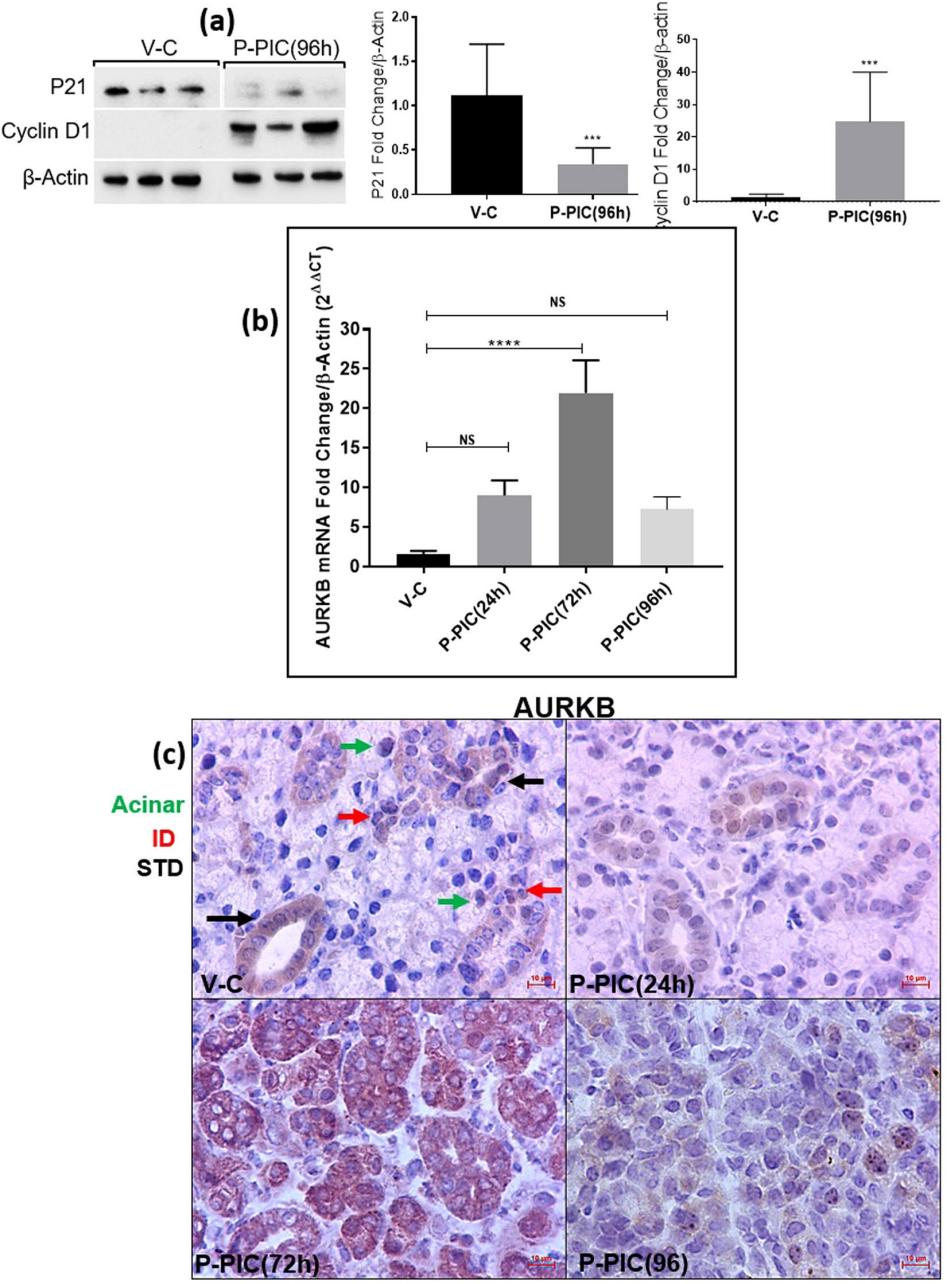
Mitogenic signals during SMG regeneration. (**a**) Extremely significant downregulation of the cell cycle repressor p21^CIP1^ and upregulation of cyclin D1 (mean ± SD, n ≥ 4, ***P < 0.001). The western blots represent samples from three independent animals in each group and the cropped blots obtained by each protein evaluation. Loading control (β-actin) was run on the same blot. All gels/blots were run in the same experimental conditions. (**b**) Significant transcriptional upregulation of AURKB marked early glandular recovery (72 hrs post poly I:C) (mean ± SD, n ≥ 4, ****P < 0.0001). (**c**) At the subcellular level, IHC revealed intense staining of AURKB in residual acini, ducts and duct-like structures during regeneration. Scale bar = 10 µm.

To analyse changes in the mitogenic program of the SMG during regeneration, cell-cycle regulators were assessed. During initial exocrine regeneration, the poly (I:C)-injected SMGs revealed dramatic loss of the cyclin-dependent kinase inhibitor (CDKi) p21^CIP1^ (inhibits nearly all CDKs, to maintain cells in a non-dividing, non-proliferative state^7^) and higher levels of the cell cycle marker Cyclin D1 (marks G1/S transition^8^) (Fig. 3a). In addition, initial observation from a PCR array study, identified significant upregulation of the cell cycle enhancer AURKB^9^ during SMG regeneration (unpublished data). To further investigate this novel finding, qRT-PCR was conducted and revealed an approximately 25-fold increase in AURKB, marking the initial regeneration of the poly (I:C)-injected glands (Fig. 3b). Importantly, because the SMGs at this time point (72 hrs post poly (I:C)) were heavily infiltrated with immune cells, IHC was conducted to confirm the parenchymal expression of AURKB and rule out any immune cell labelling. These experiments revealed that AURKB is expressed extensively and almost exclusively in the residual acinar cells and duct-like structures making-up the regenerating glands (Fig. 3c).

### AURKB *in vivo* blockade during SMG regeneration

**Figure 4:**
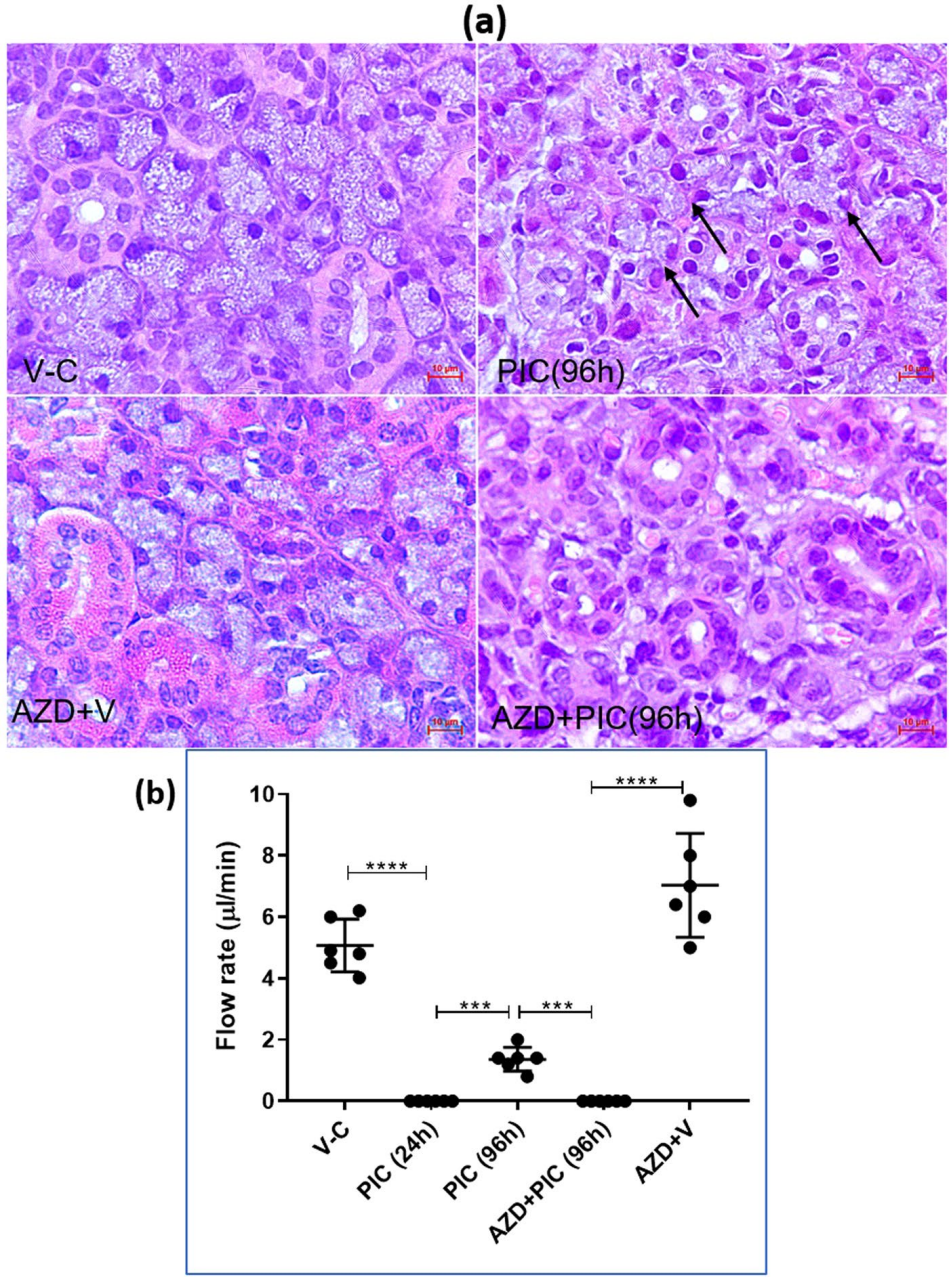
(**a**) Overall examination of H&E sections revealed a lack of acinar cells in the poly (I:C)-injected glands after AURKB blockade, in contrast to the newly emerging cells (arrows) that were clearly seen in the normally recovering SMGs. Scale bar = 10 µm. (**b**) Functional consequences of AURKB inhibition during SMG regeneration. Typically, 96 hrs post poly (I:C) local introduction, the SMGs exhibited significant restoration of salivary secretion. Conversely, at the same time point, AURKB inhibition resulted in sustained loss of salivary secretion, similar to that seen 24 hrs post immune challenge (mean ± SD, n ≥ 6, ***P < 0.001, ****P < 0.0001).

Aurora B kinase has been characterized as a novel subfamily of serine/threonine kinases that play indispensable roles in the control of cell division. To evaluate whether interference with AURKB activity will undermine SMG regeneration, a highly selective pharmacological inhibitor of AURKB activity (AZD1152-HPQA/Barasertib) was used. On the day of retroductal injection of poly (I:C) or the vehicle, mice received 25 mg/kg *i*.*p*. AZD1152-HPQA and for three more consecutive days (total of 4 *i*.*p*. doses)^10^. Saliva and tissue sections were collected 96 hrs following the intraductal infusions (Supplementary Fig. S7a). Gross examination of the SMGs from the AZD1152-treated and non-treated animals revealed considerable shrinkage in the glands which dually received local poly (I:C) and systemic Barasertib (Supplementary Fig. S7b), an observation which provisionally indicated less cellularity/replenishment of the poly (I:C)-injured glands. Indeed, histologic examination of the poly (I:C)-injected glands demonstrated absence of acinar cells in response to AURKB inhibition (Fig. 4a). One very important consequence of blocking AURKB during early SG regeneration was the sustained loss of saliva production (Fig. 4b).

### Cell cycle disruption and inhibition of mitosis in response to AZD1152-HPQA

**Figure 5:**
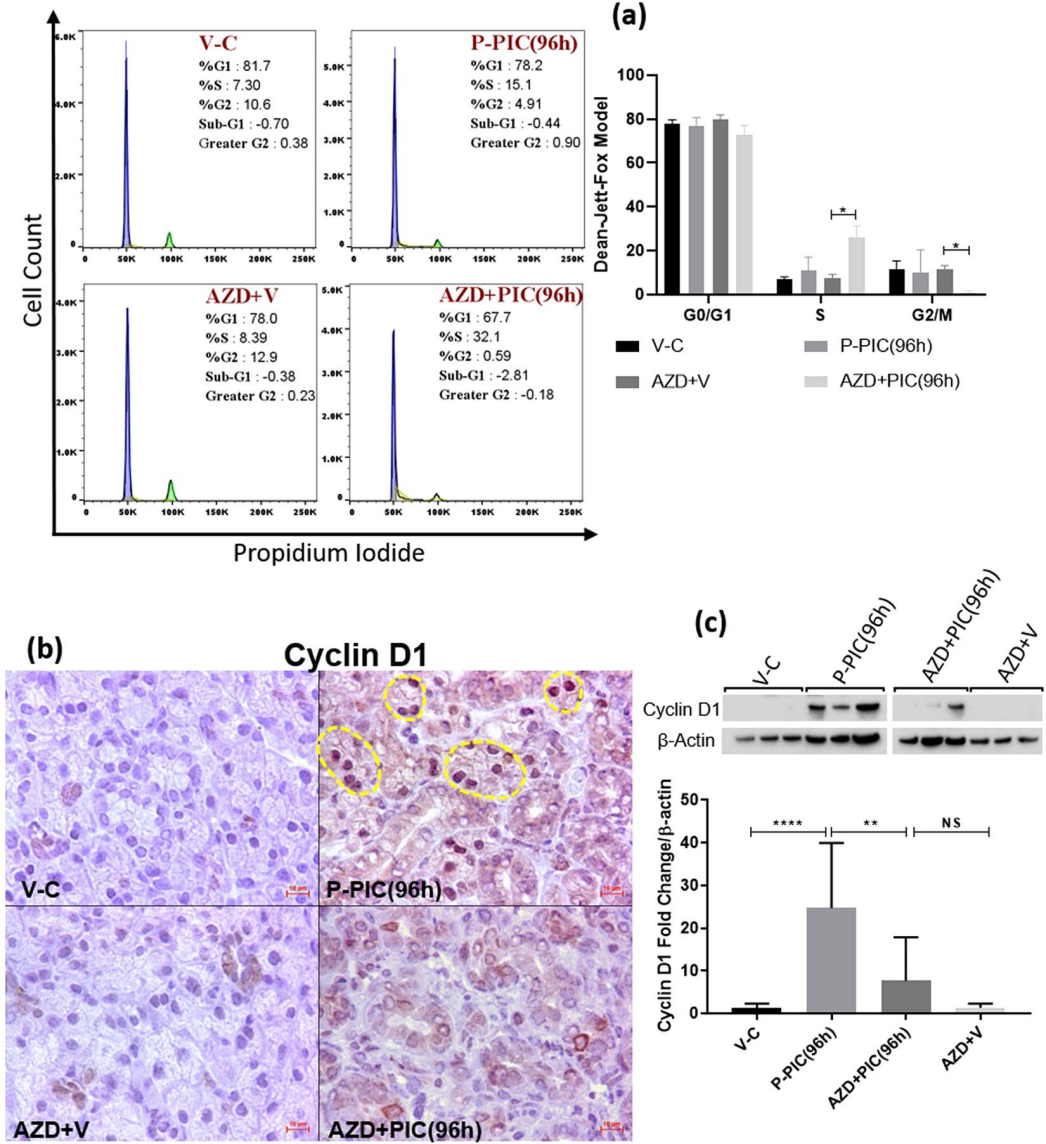
(**a**) AZD1152-HQPA-dependent cell cycle modification. SMG single cells from AZD1152-treated and non-treated animals were stained with propidium iodide/RNAse solution and analysed by flow cytometry. Data shown are representative of three independent experiments, ((n = 3), *p < 0.05). (**b**) IHC images of cyclin D1-positive cells showing sporadic nuclear expression in the control glands. Poly (I:C)-induced high expression of cyclin D1, mainly in acinar nuclei (outlines). Conversely, AURKB inhibition interfered with cyclin D1 upregulation. Scale bar = 10 µm. (**c**) SMG lysates showing remarkable loss of cyclin D1 in response to AURKB blockade (mean ± SD, n ≥ 3, **P < 0.01, ****P < 0.0001 and NS: non-significant). The figure represents samples from three independent animals. Loading control (β-actin) was run on the same blot. All gels/blots were run under the same experimental conditions.

**Figure 6:**
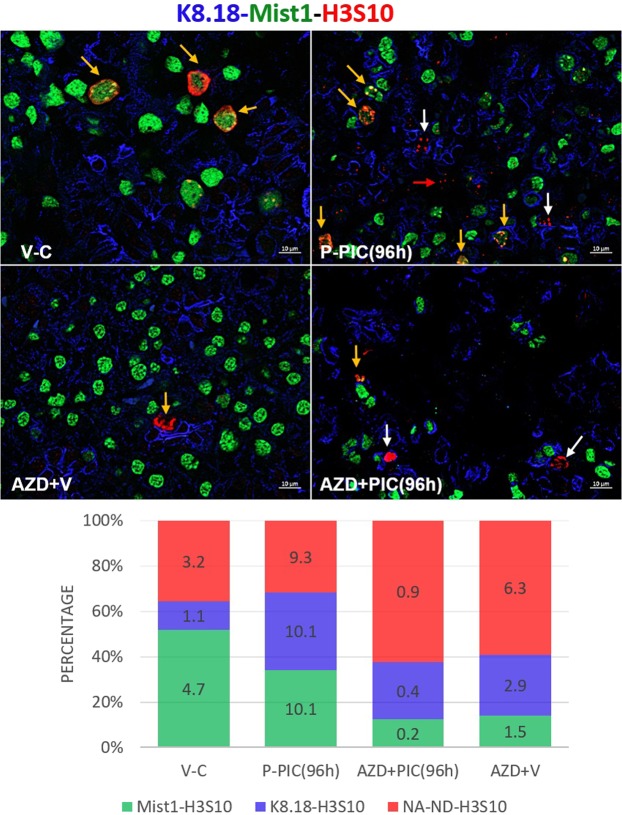
AURKB blockade interfered with mitotic division of the SMG cells during regeneration. Low-grade mitosis was recorded in the control SMGs, whereby acinar cells showed higher mitotic activity (yellow arrows), compared to the other compartments, including duct cells. At 96 hrs post poly (I:C), Mist1-k8.18-H3S10 co-labelling revealed equal levels of enhanced mitosis taking place in the tested compartments. Note the overall dramatic reduction in the average number of dividing cells (figures inside the stacked columns), perceived with AURKB inhibition. In addition, in the absence of poly (I:C), AZD1152 caused considerable reduction in the number of cells double positive for H3S10 and Mist1 (orange arrows), in contrast to relatively higher mitosis of the ductal cells (white arrows) and other non-acinar, non-ductal cells (red arrow). Note that AZD1152-maintained comparable percentages of cells labelled with H3S10, in the presence and absence of poly (I:C). Scale bar = 10 µm.

 Considering these results and the well acknowledged role of AURKB in promoting the cell cycle and mitosis, we hypothesized that AZD1152 interfered with normal re-cycling of SMG cells to repopulate the lost compartments. Flow cytometry was conducted to validate our hypothesis and investigate the effects of AZD1152-HQPA on SMG cell cycle progression. As shown in Fig. 5a, poly (I:C) local introduction nearly doubled the number of proliferating cells (S-phase): from 7.3% in the control to 15.1% in the immune-challenged glands. On the other hand, AZD1152-HQPA treatment resulted in significant accumulation of SMG cells within the S-phase, parallel to lack of cells entering the G2/M phase (gating strategy is detailed in Supplementary Fig. S8). To further confirm this general overview of AZD1152-mediated cell cycle arrest/disruptions, experiments were conducted to assess levels of the cell cycle marker Cyclin D1. The selective AURKB inhibitor interfered with the parenchymal cell cycle, evidenced by the very significant loss of cyclin D1 in the stained tissues (Fig. 5b) and tissue homogenates (Fig. 5c). Since phosphorylation of H3 at the S10 residue is a well-acknowledged event marking AURKB activity and entry of cells into mitosis (27), the overall progression of AZD1152-treated and non-treated, SMG cells into mitosis was assessed by co-localizing H3S10 with ductal, acinar and progenitor markers. Consistent with the flow cytometry results, AURKB blockade noticeably diminished the total number of mitotically-active, H3S10-positive cells during recovery from poly (I:C) (AZD1152 + PIC(96 h)) (Fig. 6 and Supplementary Fig. S9). Importantly, the worst mitotic deficiency was perceived in the Mist1-positive population, not only in the poly (I:C)-injured glands, but also in the non-injured glands from animals which received the systemic AURKB inhibitor and the locally injected vehicle (AZD + V).

### Acini depend on AURKB to recover from poly (I:C)-induced depletion

**Figure 7:**
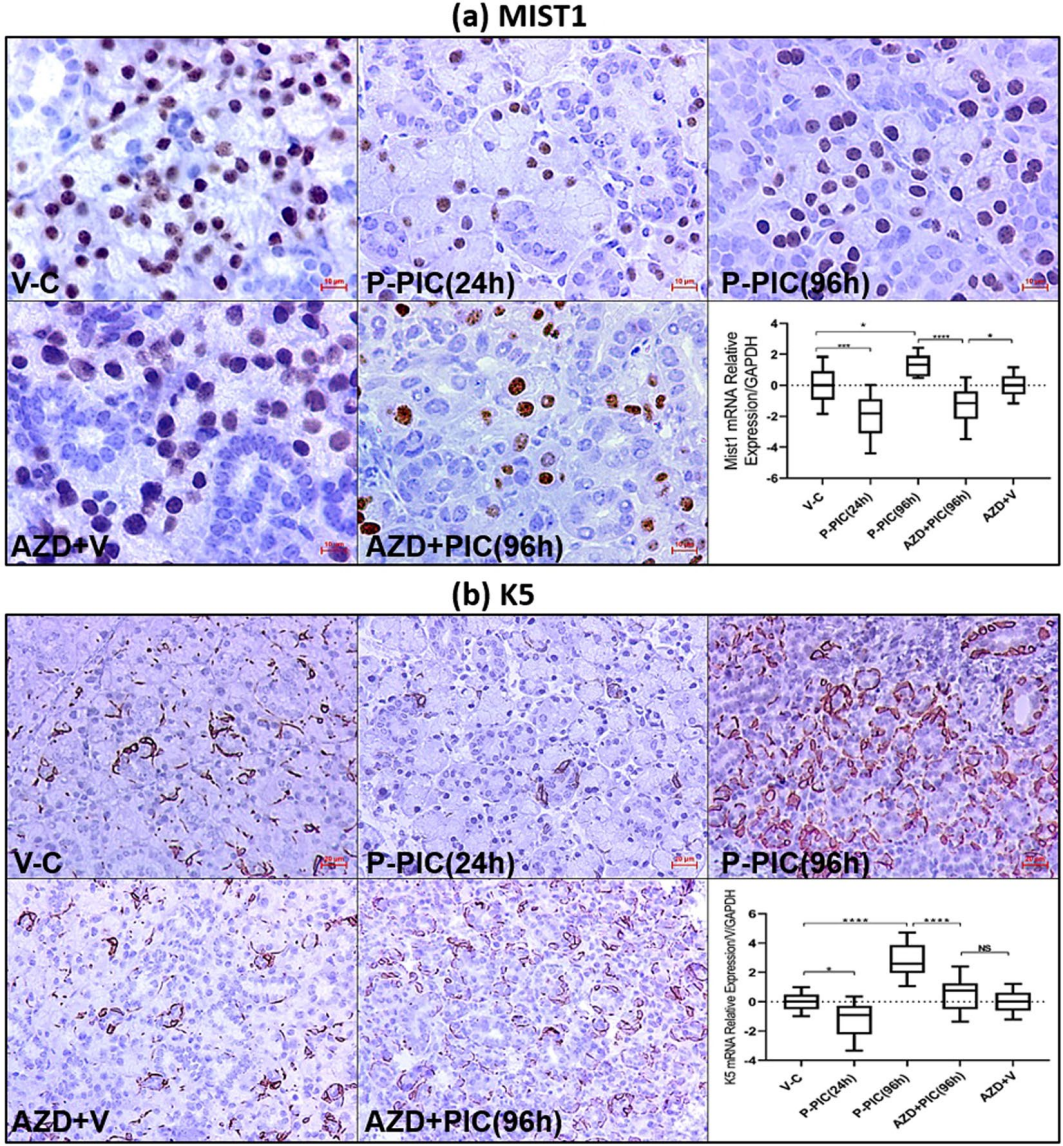
(**a**) Effect of AURKB inhibition on renewal of depleted Mist1-positive acinar cells. The detrimental effect of AURKB inhibition on acinar renewal was confirmed with Mist1 staining. AURKB selective inhibition seized Mist1 transcriptional and protein levels in an injury-like status in the SMGs challenged with poly (I:C). Scale bar = 10 µm. (**b**) Effect of AURKB blockade on progenitor expansion. The K5^+^ progenitor compartment did not show similar dependence on AURKB for replenishment and expansion. Note that AZD1152-HPQA partly prevented the extensive expansion of K5 progenitors, 96 hrs post poly (I:C), in contrast to the Mist1-sustained depletion. Scale bar = 20 µm.

To further confirm that AURKB upregulation was the key event in driving mainly acinar replenishment, IHC and PCR were conducted to compare the effects of AURKB inhibition in the acinar versus the K5 progenitor cells. These experiments demonstrated that while AURKB blockade captured Mist1 acini in the depleted, injury-like level (24 hrs post poly (I:C)) (Fig. 7a), it only interfered with the extensive expansion of K5 progenitors during SMG regeneration (Fig. 7b).

## Discussion

In this study we used a murine model to characterise early exocrine SG regeneration. Our current work highlighted the cycling plasticity and inherent ability of the adult murine SG to repopulate its lost functional compartment, following transient acute innate immune-mediated injury. In addition, our results demonstrated AURKB-driven events, that have not been previously described during SG regeneration. This work demonstrates that the selective AURKB inhibitor AZD1152-HPQA interfered with normal progression through the cell cycle phases. Also, co-localization experiments have demonstrated that the AURKB-mediated mitotic arrest dramatically affected the acinar compartment more than the ductal or K5-progenitor cells.

The innate immune stimulant poly (I:C) was used to induce injury in mouse SMGs, due to the well-documented detrimental signals downstream from its binding to the pattern recognition receptor TLR3, which comprise of: NF-ĸB-mediated cytokine release, interferon overproduction and induction of death signals^11^. Following a transient injury phase, SMGs swiftly recovered at the functional and the structural levels and this rapid response permitted accurate monitoring of the earliest events occurring and driving exocrine SG regeneration.

While the injury phase (up to 72 hrs) of this model featured dramatic acinar cell depletion, the SGs of these adult animals demonstrated a rapid re-population with Mist1-positive acini by 96 hrs. Upregulated expression of the cell cycle marker cyclin D1, suggested that SMG regeneration required an initial cell cycle re-entry phase, followed by a proliferation-dependent cell expansion process, evidenced by the co-localization of Mist1 and PCNA. Our results, among others^12,13^, demonstrate that exocrine acinar cells are not post-mitotic or terminally differentiated as the conventional theory proposes. Indeed, these functional cells were able to respond to injury signals by exploiting their recycling plasticity and undergoing significant changes in their proliferative activity.

Significant upregulation of the AURKB gene was detected in a qPCR array, which was initially conducted to characterise the regeneration signals induced after the innate immune injury (unpublished data). Using qRT-PCR and IHC, transcriptional upregulation and parenchymal overexpression of AURKB marked the functionally recovered but not injured SMG tissues. Therefore, AURKB as a novel regeneration signal was further considered in the current model.

**Figure 8:**
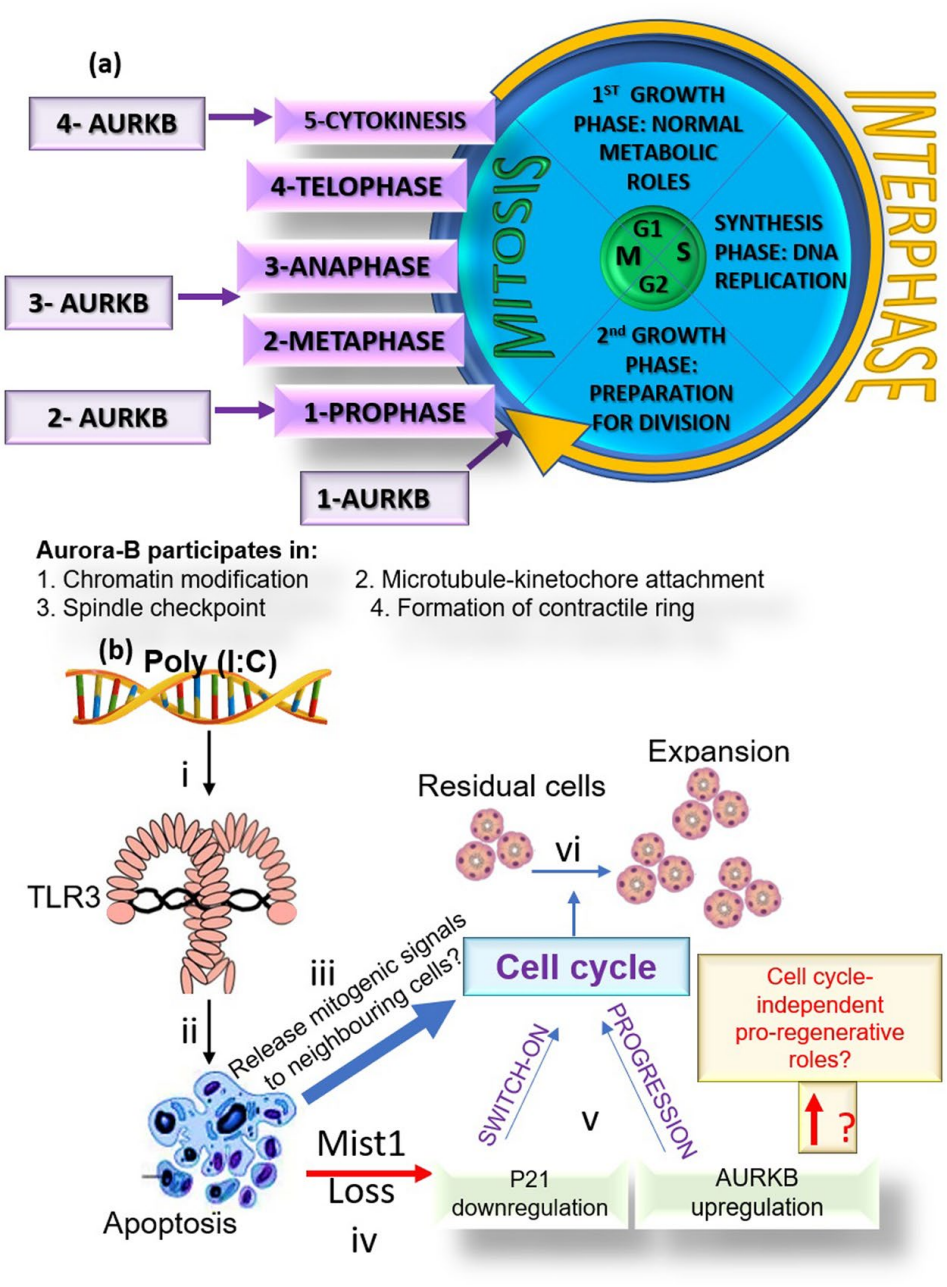
(**a**) AURKB in the cell cycle: 1- At the beginning of mitosis, AURKB phosphorylates histone H3 at the S10 residue and initiates an epigenetic switch toward an active chromatin conformation. 2 and 3- AURKB permits chromosome bi-orientation through the regulation of the spindle assembly checkpoint. The activated checkpoint detects unattached or poorly attached kinetochores. 4- AURKB induces actin polymerization, myosin activation and Vimentin, Desmin as well as GFAP phosphorylation, which are key pre-requisites for the formation of the contractile ring during cytokinesis (27). (**b**) Illustration summary depicting the verified results and hypothetical backgrounds (yellow outline and red font) of the observed findings in the current SG regeneration model. i The dsRNA mimic poly (I:C) binds TLR3 in SMGs. ii Induction of apoptotic signals. iii Dying cells may have released mitogenic signals to the neighbouring cells iv. Depletion of Mist1-positive acinar cells may have caused v re-cycling and proliferation of parenchymal cells. vi Recovered cell density and regeneration.

Aurora kinases play key roles in cell division by phosphorylating multiple targets^14,15^. AURKB comprises part of the chromosomal passenger complex (CPC)^16^, which plays an essential role in regulating mitosis and cytokinesis^17^ (Fig. 8a). In dividing cells, AURKB is responsible for phosphorylation of H3 at S10 and S28^18^, which results in chromosomal condensation and regulation of chromatin structure during early mitosis^19^. In addition, AURKB activity is indispensable for correct spindle formation, accurate chromosomal segregation and completion of cytokinesis^20^. AZD1152-HPQA is a potent and highly selective inhibitor of AURKB, acting via preventing the transfer of the phosphate group from the ATP molecule to AURKB substrates^21^. The present study demonstrated for the first time, that AURKB inhibition interferes with early regeneration of SGs. Consistent with its canonical functions, AURKB mediated its pro-regenerative effects via promoting cell cycle progression, which aided the replenishment of depleted functional cells.

Recently, it has been proposed that AURKB phosphorylation of several proteins affecting the structural organization of intermediate filaments, microtubules and actin cytoskeleton^22^, leads to regeneration of non-dividing, terminally differentiated cells like neurons^5^. The current study has clearly underscored the AURKB-promoted cell cycle and proliferation events during SMG regeneration. Nevertheless, IHC has shown cytoplasmic AURKB immunolabelling in the parenchymal cells making up the regenerating SMGs, suggesting other cell-cycle independent roles. Since the cytoskeleton plays indispensable roles in regulated exocytosis^23^ and polarized calcium signalling^24^, two key determinants in protein and fluid secretion in the SGs, AURKB cytoplasmic expression warrants further investigation.

It remains unclear what are the signalling mechanism(s) which lead to the activation of AURKB downstream of poly (I:C) stimulation . Two potential mechanisms can be involved in this model. The first one is related to Mist1 being a part of the bHLH family of transcription factors and assembling with other bHLH proteins such as E47. E47 has been recently shown to induce G0/G1 arrest and shape the acinar cell differentiation program by regulating key mitogenic genes such as p21, **aurora kinase**, topoisomerase and cyclin B^25^. Dramatic depletion of the Mist1 phenotype during the injury phase of the current model may have aided in switching off the regulatory p21^CIP1^^7^ and generating the cycling events and AURKB upregulation, observed during regeneration. Secondly, recent evidence has suggested that proapoptotic proteins— mainly caspases—can induce proliferation of adjacent cells, that survived the injury stimulus, to replace dying cells^26^. This apoptosis-induced compensatory proliferation and the transcriptomic alterations associated with it, has never been studied in the SGs. The poly (I:C) injury/regeneration model presented herein can be exploited to unveil the beneficial advantage of innate immune stimulation to induce proliferation and salivary cell re-cycling in chronically damaged/aged glands.

It is generally thought that salivary gland regeneration following injury involves proliferation of progenitor/stem cell populations, which have been localized to intercalated and main excretory ducts, followed by differentiation and replenishment of the secretory acinar cell population (Man *et al*., 2001; Pringle *et al*., 2013). Earlier studies highlighting a role for acinar cell proliferation in regeneration and maintenance of glandular homeostasis in mouse and human (Denny *et al*., 1993; Irhler *et al*., 2004) have recently been reinforced using lineage tracing (Aure *et al*., 2015) and the present study again indicates a contribution from proliferating, differentiated acinar cells. The present study also reports for the first time some key molecular events during regeneration of salivary gland epithelia. AURKB regulation of the cell cycle plays a crucial role in salivary gland functional restoration and structural re-population, following transient innate immune-mediated injury and acinar depletion.

To conclude, this study reports for the first time key molecular events during recovery of SG epithelia. AURKB, which is highly characterised within the context of cancer cell division, played a vital role in SG functional restoration and structural re-population, post transient innate immune-mediated injury and acinar depletion. Figure 8b depicts the reported facts and future questions that can be answered using the recently introduced SG regeneration model.

## Materials and Methods

### Mice

Female C57BL/6 mice weighing 18–21 grams (Harlan Labs Ltd., Loughborough, UK) and aged 10–12 weeks were housed in a temperature-controlled environment under a 12 h light–dark cycle, with free access to food and water. All procedures were approved by King’s College London Animal Welfare and Ethical Review Body (AWERB) Committee and performed under general anaesthesia under a Home Office license. All methods were performed in accordance with the relevant guidelines and regulations of King’s College London.

### Retroductal injections

The C57BL/6 mouse SMGs were cannulated as previously described^1^. Mice were anesthetized with intraperitoneal *(i*.*p*.*)* injection of 0.1 ml combined ketamine (100 mg/kg) and xylazine (10 mg/kg). SMG ductal cannulation was performed using 0.3 ml syringe (6134900, VWR International) attached to a glass cannula (Supelco, 25715, PA- USA), which was inserted into Wharton’s duct under a stereomicroscope (Fig. 1a). Poly (I:C) (P1530-25MG, Sigma-Aldrich) (4 mg/ml) was injected slowly and constantly into both SMGs. The same volume of the vehicle was delivered to the SMGs of the negative control group.

### Saliva collection and analysis

For functional assessment, mice were anaesthetized with 150 µl of pentobarbital sodium (Euthatal, Merial) 1 mg/ml (i.p.), followed by endotracheal intubation. SMG ducts were ventrally exposed and cut. Saliva was collected via polyethylene tubes in pre-weighed Eppendorf tubes. Saliva collection proceeded for 5 min following stimulation with pilocarpine (0.5 mg/kg *i*.*p*.). The volume of saliva was calculated as 1 mg = 1 μl saliva and results were expressed as µl saliva/min.

### Histology, immunohistochemistry (IHC) and digital image analysis

Harvested SMGs were fixed in 10% neutral buffer formalin, processed and embedded in paraffin for long term storage. Histomorphological changes were examined using conventional H&E stain. For immunohistochemical studies, 3 µm tissue sections were deparaffinized, rehydrated, and unmasked in a single step using Trilogy™ (Cell Marque, Rocklin, CA, 920P-06). To block endogenous peroxidase activity and non-specific background staining sections were incubated in 3% hydrogen peroxide solution for 20–30 minutes. To block all epitopes on the tissue samples and prevent nonspecific antibody binding, sections were incubated with 1% BSA in 1X TBS, pH 7.6 for 5 minutes. Tissue sections were incubated at 4 °C overnight, with the antibody: rabbit-anti-cleaved caspase 3 [1:2500, NB100-56113, Novus Bio], rabbit–anti-Mist1 [1:200, ab187978, Abcam], rabbit-anti-Cyclin D1 [1:600, 2922S, Cell Signalling Technology], rabbit-anti-AURKB [1:200, ab2254, Abcam], mouse-anti-PCNA [1:2000, 2586, Cell Signalling Technology], mouse-anti-H3S10 [1:1000, ab14955, Abcam], goat-anti-AQP5 [1:100, sc-9891, Santa Cruz Biotechnology], rabbit-anti-NKCC1 [1:6000, ab59791, Abcam], rabbit-anti-c-Kit [1:300, 3074, Cell Signalling Technology], rabbit-anti-K5 [1:2000, PRB-160P, Biolegend], rabbit anti-Sox9 [1:500, AB5535, Millipore], anti-guinea pig K14 [1:50, ab192694, Abcam], or anti-guinea pig K8.18 [1:50, ab194130, Abcam], followed by 1 hr incubation with the goat anti-rabbit (1:200, P0448, Dako), goat anti-mouse [1:500, A32723, Invitrogen], goat anti-rabbit [1:500, A27039, Invitrogen], donkey anti-goat [1:500, A11057, Invitrogen] or goat anti-guinea pig [1:500, ab175678, Abcam]. For peroxidase reactions, colour was developed for 5 mins in DAB solution (Pierce™ 34002) and slides were counterstained in Mayer haematoxylin and DPX-mounted for light microscopy. Sections for immunofluorescence imaging were mounted using either: VECTASHIELD Antifade Mounting Medium with DAPI (Vector Laboratories), or aqueous mounting medium, ab128982, Abcam) and imaged using a ZEISS Apotome microscope with ZEISS ZEN imaging software. For cell count and area percentage analysis: fifteen, random, 20x/40x magnification fields (≥5 fields from three independent experiments) were captured and color images of 640 × 480 pixel resolution were then analyzed by semi-quantitative digitalized image analysis using ImageJ, NIH®. For Mist1-positive cell count and area percentage of CK14, values are demonstrated as mean ± standard deviation (S.D.).

### Analysis of cell proliferation and mitosis

The percentage of Mist1^+^/PCNA^+^, K8.18^+^/PCNA, K5^+^/PCNA^+^, Mist1^+^/H3S10^+^ and K8.18^+^/H3S10^+^ double positive cells were calculated by counting the total number of cells showing double positivity divided by the total number of either PCNA^+^, or H3S10^+^ cells. Values are demonstrated as means in 100% stacked column charts for the PCNA experiments and means in stacked column chart for the H3S10 experiment.

### Western blot

Tissues stored in RNAlater® were retrieved, homogenized in cell lysis buffer (AA-LYS-10 ml- RayBiotech, Inc., Norcross, GA) plus protease inhibitor cocktail (1:10 dilution, Calbiochem, UK) using a FastPrep™ tissue homogenizer (MP Biomedicals Santa Ana, CA). Protein concentration was measured using the Qubit® protein assay kit (Q33211, Invitrogen™, UK) and Qubit® 3.0 Fluorometer (Q33216, Invitrogen™, UK) and a total of 15–20 μg/lane of the different lysates were separated by SDS-PAGE on a 4–12% Novex polyacrylamide gel (Invitrogen, UK). Electro-transfer of proteins was done for 1 hour to 0.2 µm pore–size nitrocellulose membrane (1620112, Bio-Rad, UK) according to standard protocol (Invitrogen, UK, Paisley), followed by membrane blocking with 5% bovine serum albumin. Membranes were incubated at 4 °C overnight with the rabbit-anti-cleaved caspase 3 [1:5000, NB100-56113; Novus Bio], rabbit-anti-p21 antibody [1:1000, [EPR18021] (ab188224); Abcam], rabbit-anti-Cyclin D1 [1:600, 2922S, Cell Signalling Technology], in blocking buffer then washed and incubated with the HRP conjugated goat anti-rabbit (1:200, P0448; Dako) in blocking buffer at room temperature for 1 h. For signal development, an Enhanced Chemioluminescence substrate (ECL, GE Healthcare, UK) was prepared following the kit manufacturer’s recommendations and applied over the membranes. Positive and negative protein expression was assessed and captured using ChemiDoc™ MP System (Bio-Rad, UK).

### qRT-PCR

For RT-qPCR analysis, SMGs stored in RNAlater® (R0901-100ml, Sigma-Aldrich) were homogenized using a FastPrep™ tissue homogenizer (MP Biomedicals Santa Ana, CA) and RNeasy® Micro Kit (74004, Qiagen) was used for total RNA extraction. RNA concentration as well as the A260/280 and A260/230 ratios were then measured with the NanoDrop ND-1000 Spectrophotometer (Thermo Fischer Scientific, Nottingham UK). iScript™ cDNA Synthesis kit (170-8890, Bio-Rad) was used to reverse transcribe 100 ng of extracted RNA. PCR reactions (10 µl/well) were prepared by adding SsoAdvanced ™ Universal SYBR Green Supermix (172–5271, Bio-Rad), primers (PrimerDesign™, Ltd. mouse AURKB, K5, c-Kit, AQP5, NKCC1, ANO1, β-Actin or GAPDH) and cDNA template. Thermal cycling was performed using Corbett RotorGene 6000 System (Qiagen, UK). In all RT-qPCR experiments, relative gene quantification was assessed according to the following equation: ΔΔCT = [Ct GOI Exp − Ct HKG Exp] − [Ct GOI Cal − Ct HKG Cal]: Ct: cycle threshold, GOI: gene of interest, Exp: poly (I:C)-injected glands, HKG: housekeeping gene, Cal: control glands injected by the vehicle. There were 3–4 biological replicates for each experiment and the values were analysed by GraphPad Prism Version 7 (GraphPad software, USA).

### AZD1152-HPQA and AURKB *in vivo* inhibition

AZD1152-HPQA (25 mg/kg, SML0268-10MG, Sigma) was given to mice by intraperitoneal injection for 4 consecutive days^10^, starting from the day of poly (I:C) retrograde injection (Supplementary Fig. S7a). Control mice received AZD1152-HPQA *i*.*p*. and local intraductal injection of vehicle (0.9% saline).

### Flow cytometry and cell cycle analysis

Single SMG cells were filtered through a 70 µ nylon sieve, washed twice with PBS and fixed in ice-cold 70% ethanol for two hours at 4 °C. Cells were washed twice with PBS and centrifuged (1000 g for 5 min). Cells were labelled with propidium iodide (PI)/RNase staining solution (4087, Cell Signalling Technology) for at least 30 minutes. Cell cycle profiles were determined using with a FACSCalibur CantoII, and data were analysed using Flow Jo™ software. Each sample was analysed in triplicate.

### Statistics

All statistical analyses were performed using GraphPad Prism 7.0 software. Statistical significance of three or more independent experiments was determined using Student t-test. One-way analysis of variance (ANOVA) with appropriate post hoc testing to correct for multiple comparisons, was used to assess significant differences (p < 0.05) between means. Two-way ANOVA with multiple comparison test was used to analyse flow cytometry and cell cycle data.

## Supplementary information

Salivary glands require Aurora B Kinase for regeneration after transient innate immune-mediated injury.

## Data Availability

All data generated or analysed during this study are included in this published article.
