# Supplementary material for: Salivary glands require Aurora Kinase B for regeneration after transient innate immune-mediated injury
Source: Sci Rep. 2019 Aug 5;9:11339. doi: 10.1038/s41598-019-47762-9 (PMC6683207; doi:10.1038/s41598-019-47762-9)
Supplement: Supplementary file 1 — Salivary glands require Aurora B Kinase for regeneration after transient innate immune-mediated injury. [file 41598_2019_47762_MOESM1_ESM.pdf]

Salivary glands require Aurora B Kinase for regeneration after transient innate immune-mediated injury.

Abeer Shaalan[1-2] and Gordon Proctor[1]

[1] Centre for Host-Microbiome Interactions, King's College London, United Kingdom

[2] Corresponding author: Abeer Shaalan, Centre for Host-Microbiome Interactions, Floor 17 Tower Wing, King's College London, Guy's & St Thomas' Hospitals, Great Maze Pond, London, SE1 9RT, shaalan.abeer@kcl.ac.uk**.**

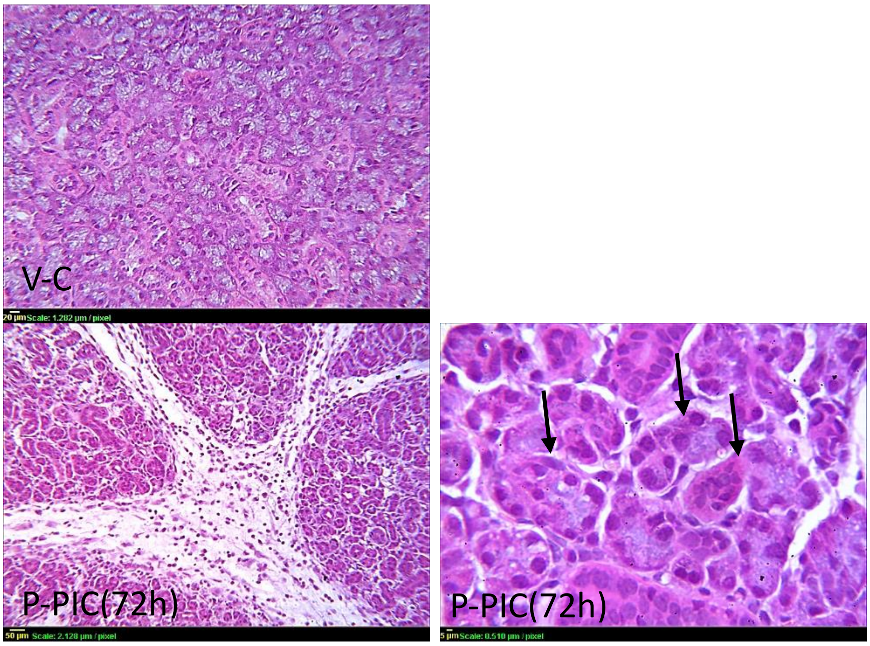

Supplementary Figure S1: Disproportionate expansion of immune cell-infiltrated, interlobular stroma, 72 hrs post poly (I:C). Lobules exhibited abundance of ducts and duct-like structures and lack of apparent acinar cells. Careful examination of high magnification images at this time point revealed residual basophilic acinar cells in direct contact to what looked histologically like duct cells (arrows).

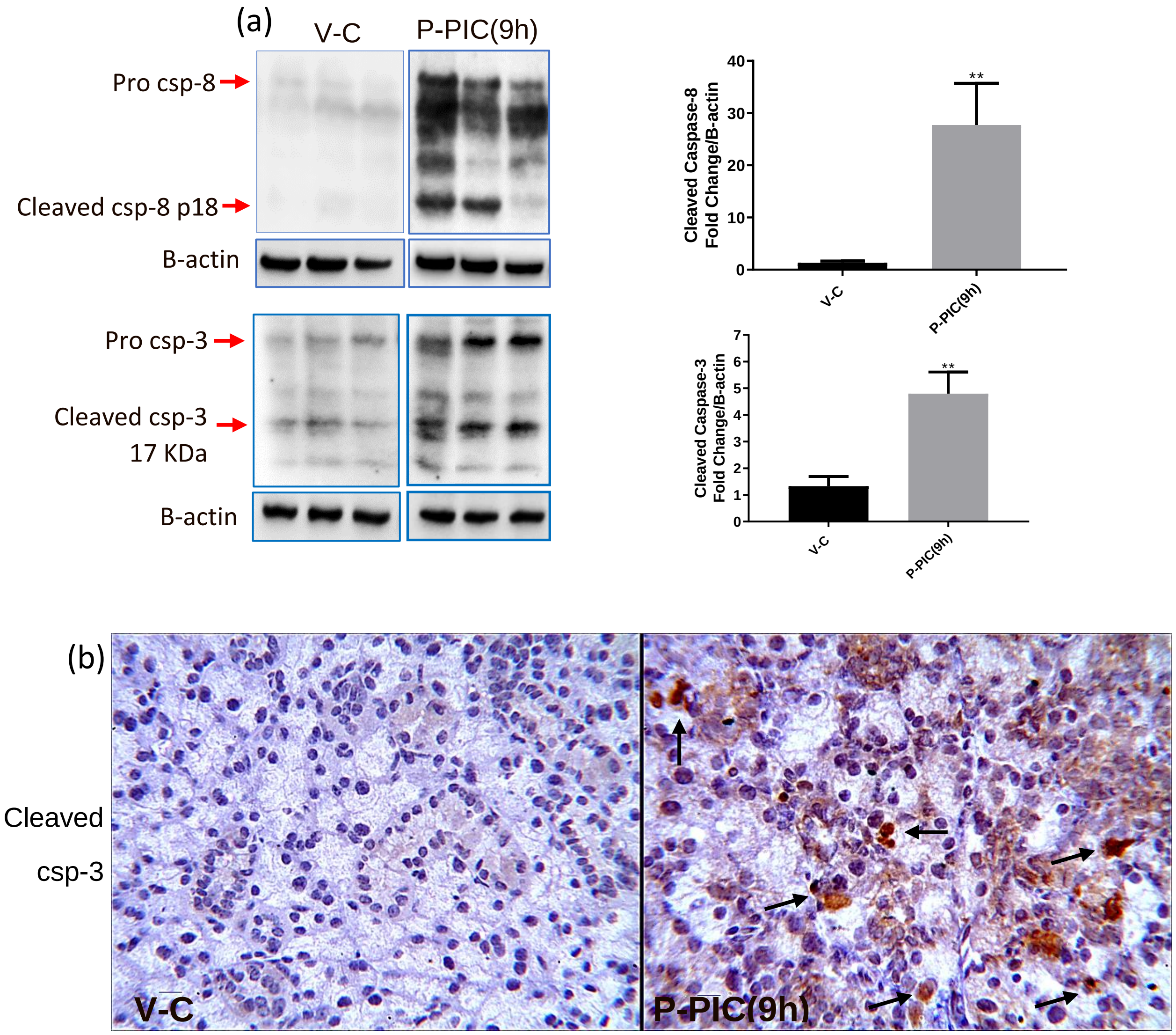

Supplementary Figure S2: Induction of apoptotic signal and acinar depletion: **a** SMG homogenates were strongly positive for the cleaved forms of caspase 8 and caspase 3 (mean ± SD, n = 3, **P<0.01). The figure represents three independent samples. Loading control (β-actin) was run on the same blot. All gels/blots were run under the same experimental conditions. **b** Immunohistochemical visualization of the SMGs before (V-C) and 9 hrs after poly (I:C) local injection. Note the abundant expression of the cleaved form of caspase 3 in cells having the histologic features of acini (arrows).

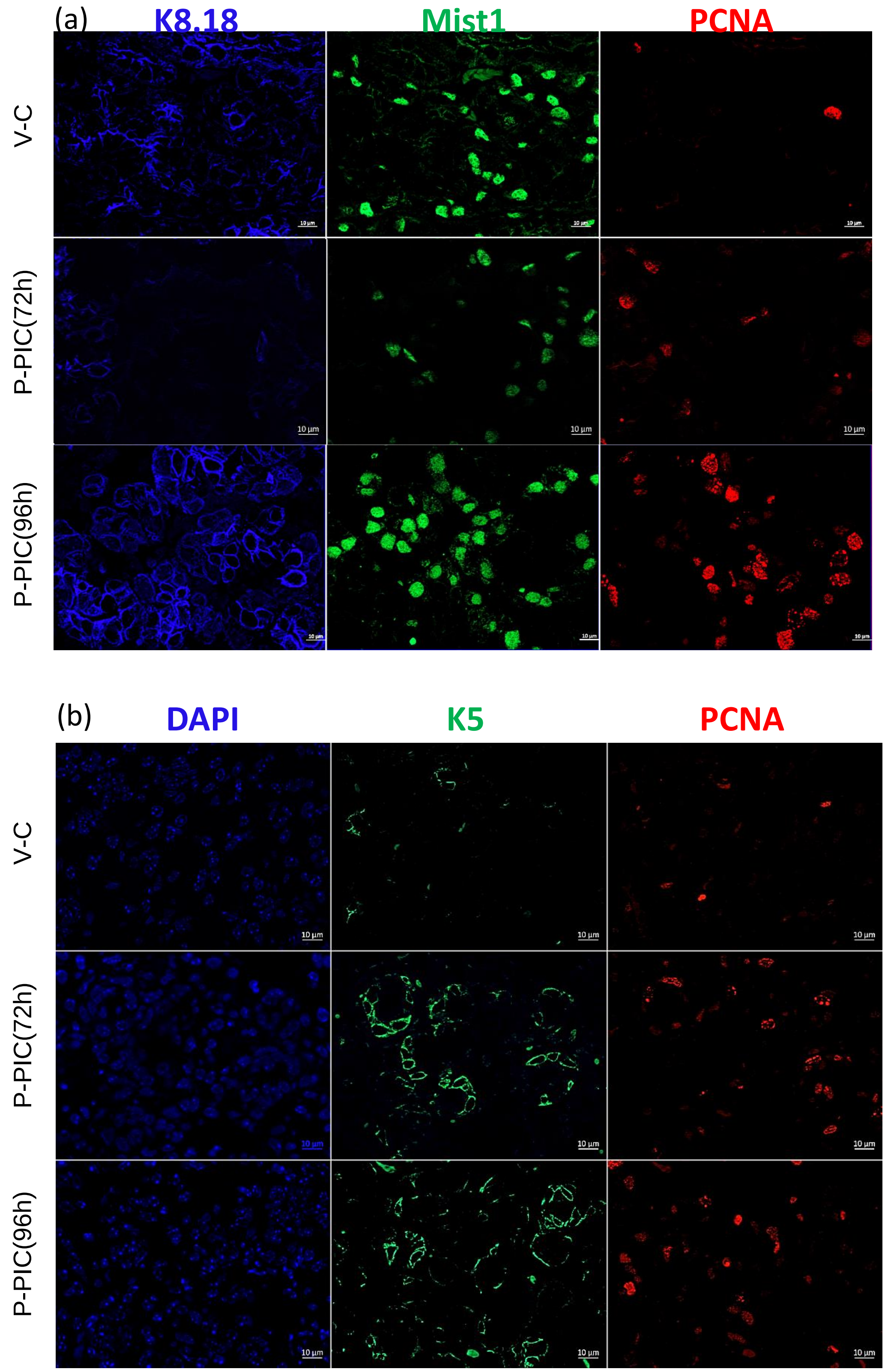

Supplementary Figure S3: a Individual channels for figure 2a in main article**. b** Individual channels for figure 2b in main article.

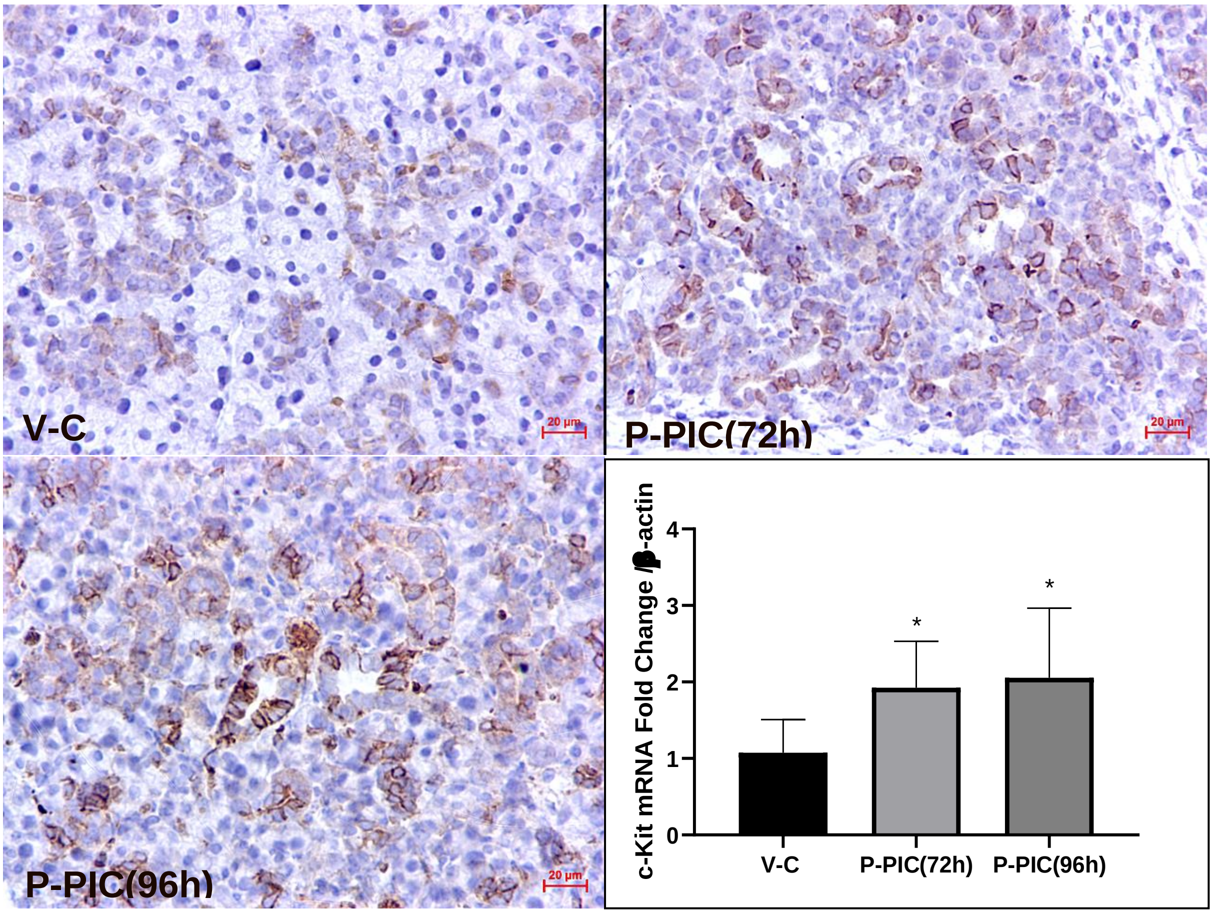

Supplementary Fig. S4 IHC and mRNA expression of the stem/progenitor marker c-Kit showing increase in the number and transcriptional activity of these cells in response to poly (I:C) stimulation (mean ± SD, n = 3, *P<0.05). Scale bar= 20 µm.

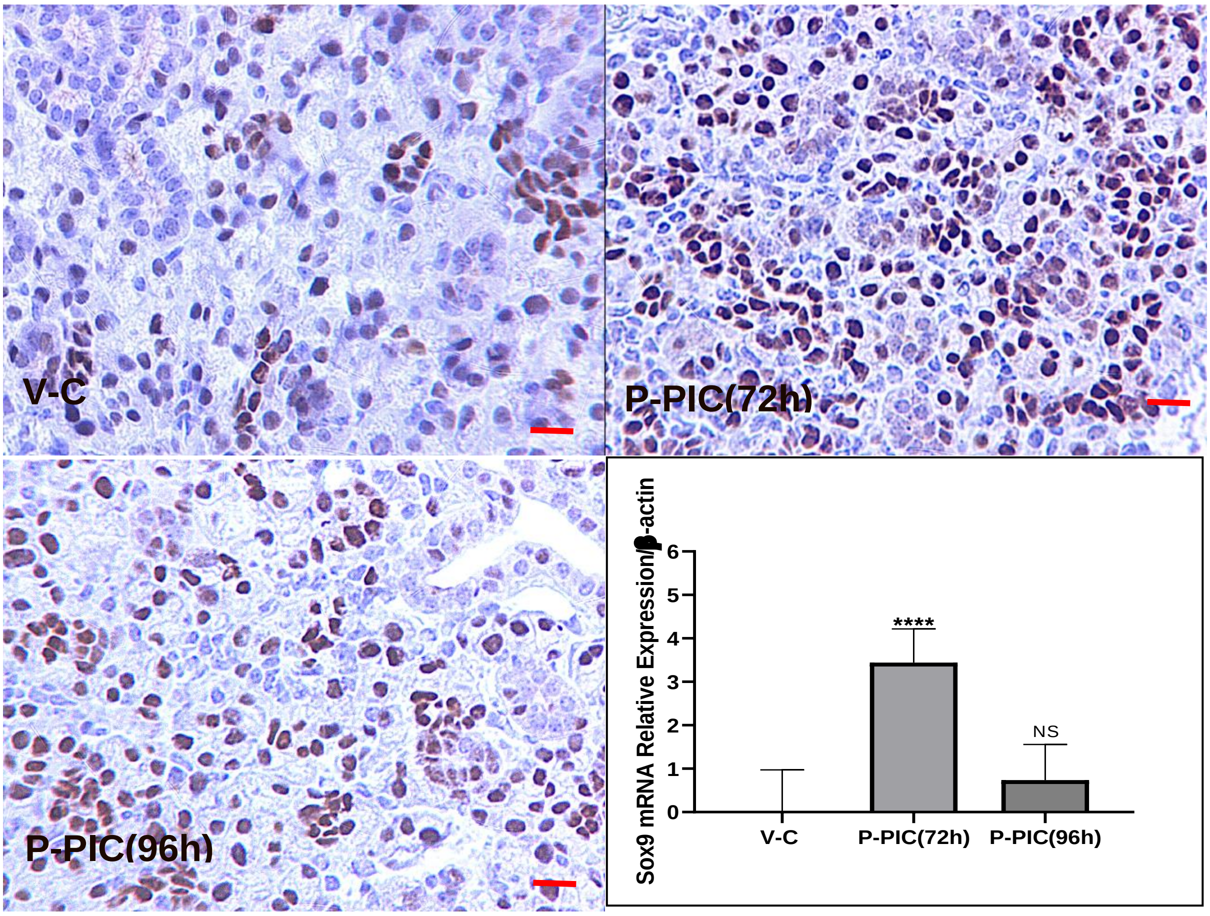

Supplementary Fig. S5 IHC and mRNA expression of the progenitor marker Sox9 showing poly (I:C)-triggered expansion and extremely significant transcriptional activity of these cells at 72 hrs post poly (I:C). While the transcription was nearly back to normal at 96 hrs, abundant Sox9-positive cells were still seen in the recovered glands (mean ± SD, n = 3, ****P<0.0001, NS: non-significant). Scale bar= 20 μm.

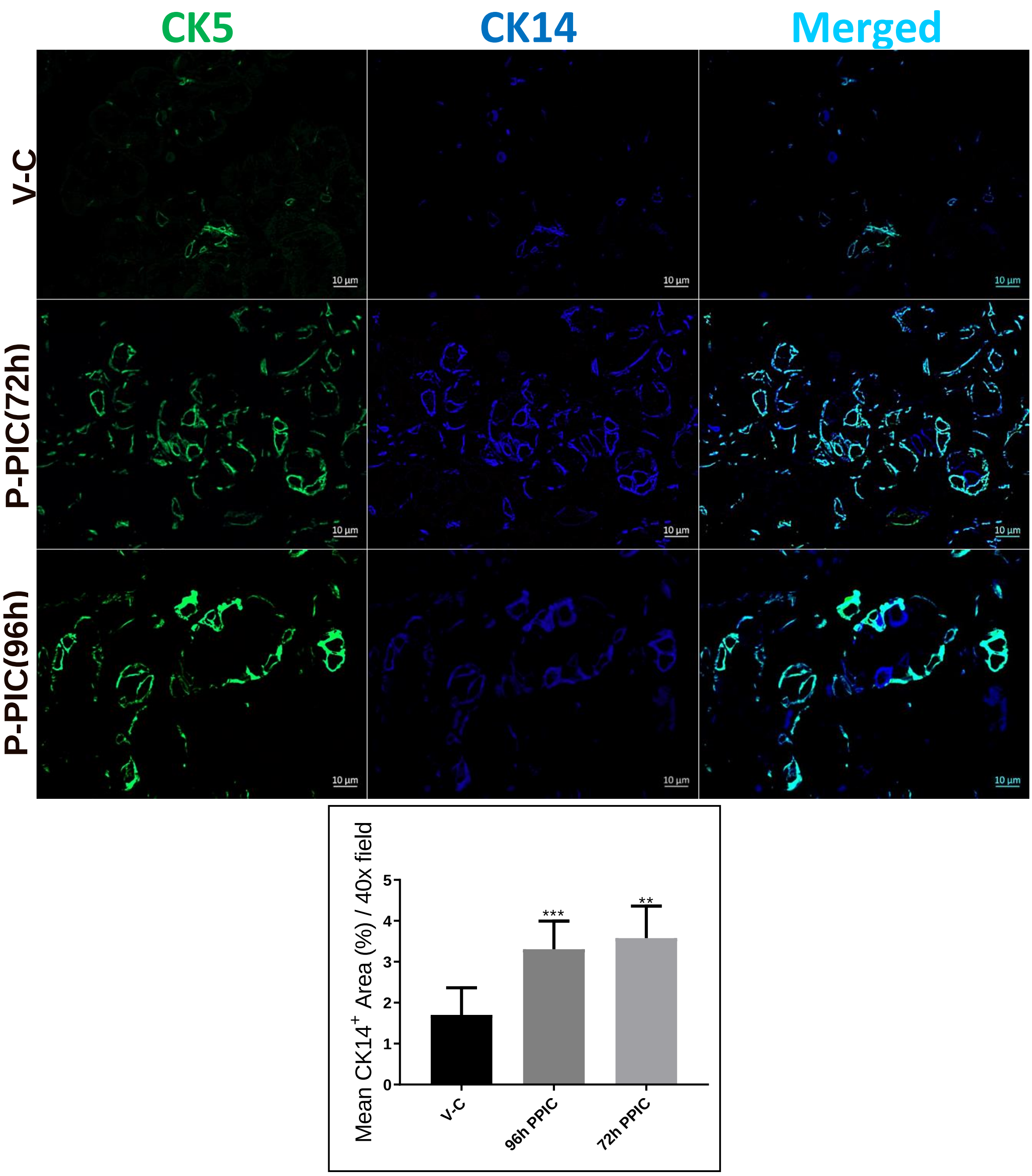

Supplementary Fig. S6 IHC and Ck14 area percentage: Increased positivity of CK14 progenitors at 72 and 96 hrs post poly (I:C). Albeit the abundant CK5-CK14 co-localisation observed, CK14 was individually spotted labelling some sporadic cells in the tested groups, (mean ± SD, n ≥15 fields (40x) from three independent animals per tested group, ***P<0.001, **p<0.01). Scale bar= 10 µm.

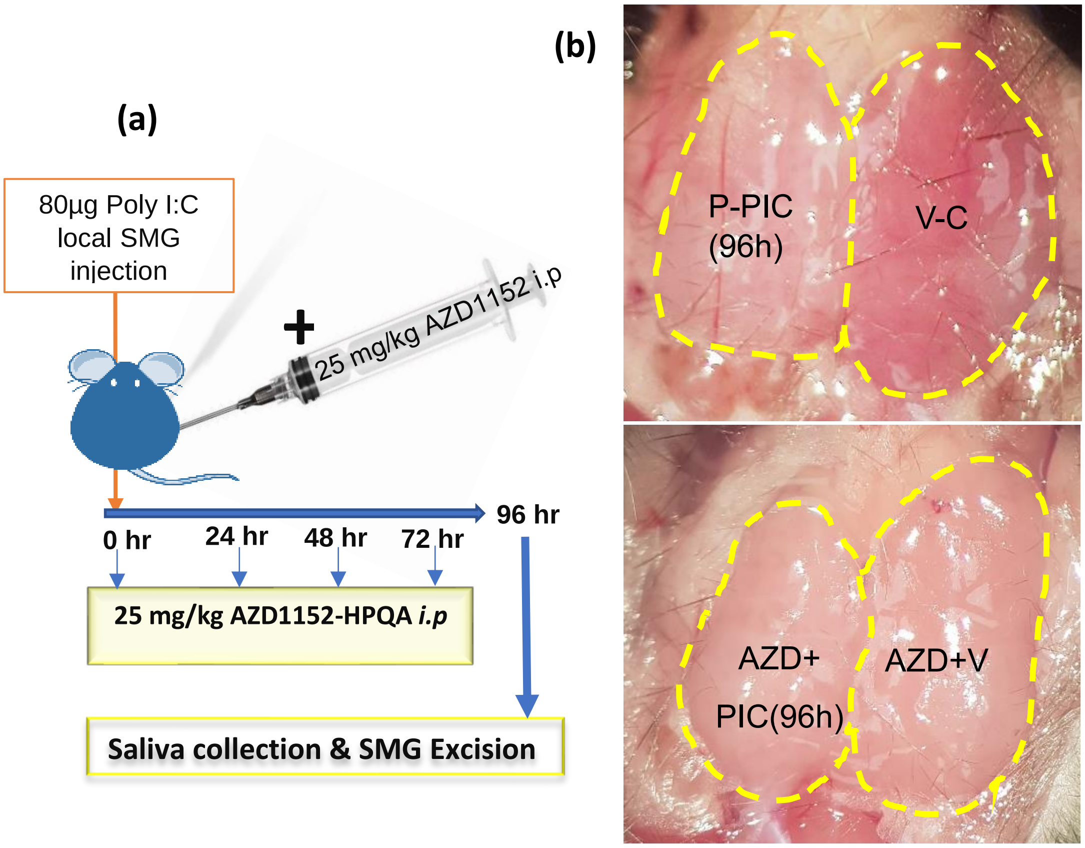

Supplementary Fig. S7 a Experimental protocol and timeline for AURKB inhibition in vivo. Four consecutive doses of 25mg/kg AZD1152-HPQA were i.p. injected, starting at the day of poly (I:C) retrograde injection (directly after SMG local infusion). Saliva and tissues were harvested at 96 hrs (timeline not to scale). **b** Gross examination of the tested glands: note the shrunken appearance of the poly (I:C)-injected glands in the mouse which received the AZD1152 inhibitor.

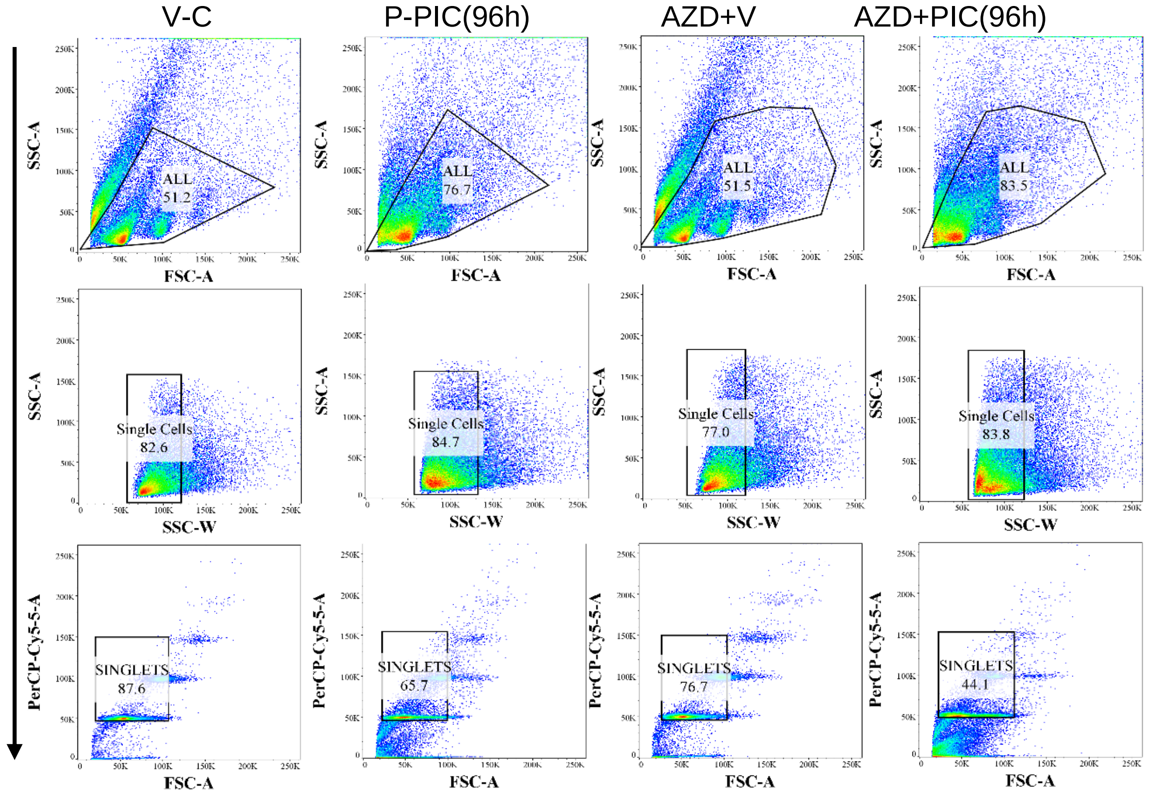

Supplementary Figure S8: Gating strategy applied to PI-stained SMG cells. First obvious debris were gated out using the forward and side scatter. Robust doublet discrimination was first done using side scatter width vs. side scatter area, followed by forward scatter vs. PI signal.

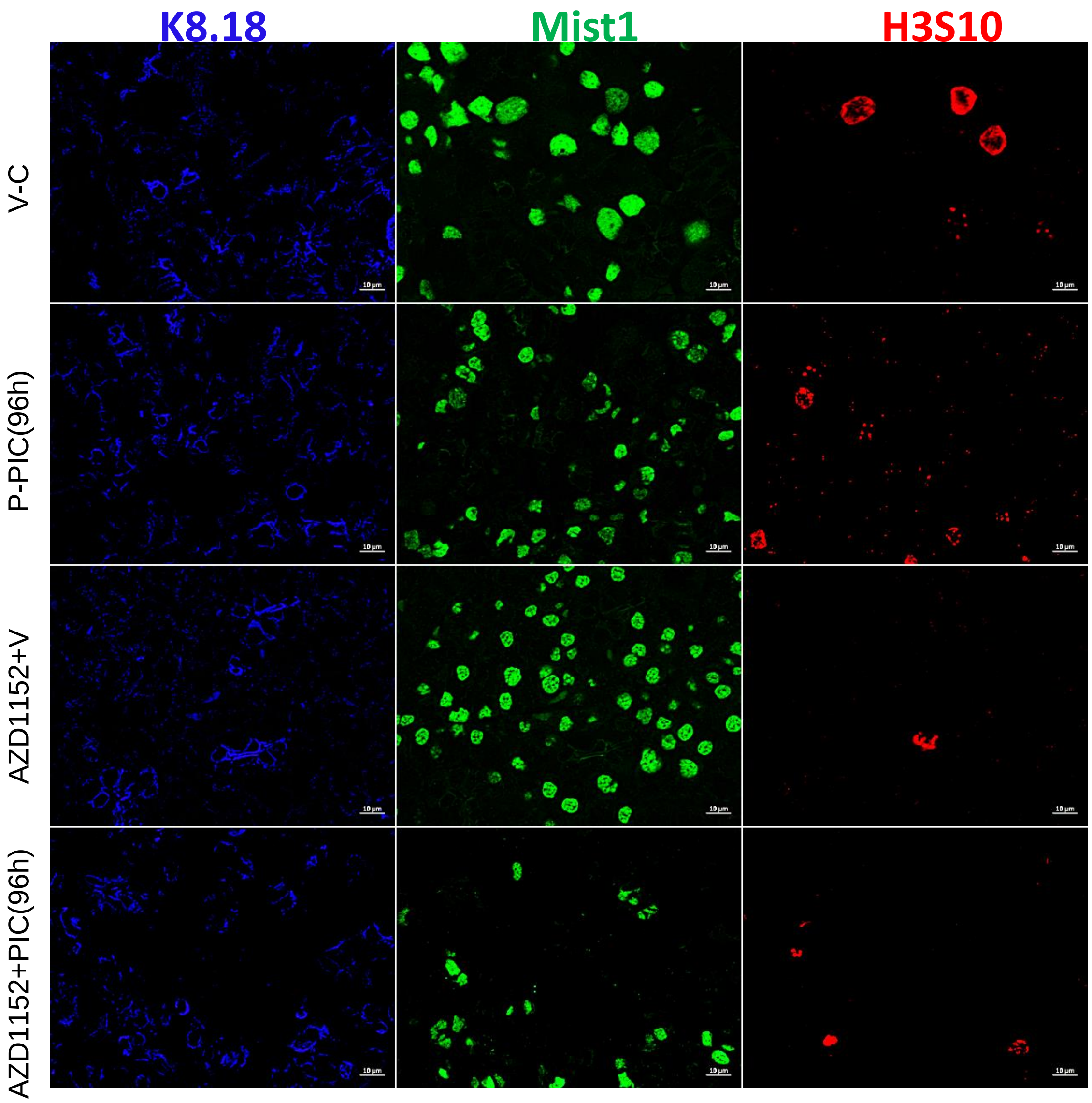

Supplementary Figure S9: Individual channels for figure 6 in main article.
